# Characterization of the ZAP-X® Peripheral Dose Fall-Off

**DOI:** 10.7759/cureus.13972

**Published:** 2021-03-18

**Authors:** Georg A. Weidlich, Woody Chung, Srejitha Kolli, Ishwarya Thirunarayanan, Thibaut Loysel

**Affiliations:** 1 Radiation Oncology, National Medical Physics and Dosimetry Company, Palo Alto, USA; 2 LINAC Development, Dosimetry, and Validation, Zap Surgical Systems, Inc., San Carlos, USA; 3 Quality Assurance, Zap Surgical Systems, Inc., San Carlos, USA; 4 Software Development, Zap Surgical Systems, Inc., San Carlos, USA; 5 Treatment Planning, Zap Surgical Systems, Inc., San Carlos, USA

**Keywords:** stereotactic radiosurgery, detector comparison, peripheral dose fall-off measurement, beam penumbra, small-field dosimetry, zap-x radiosurgical system

## Abstract

Various small-field radiation dose detectors were systematically compared and their impact on measured beam performance of the ZAP-X® dedicated stereotactic radiosurgery system (ZAP Surgical Systems, Inc., San Carlos, CA, USA) was determined. Three Physikalische Technische Werkstaetten (PTW) diodes, i.e., the microSilicon, the microDiamond, and the Stereotactic Radiosurgery (SRS) diode detectors of (PTW-Freiburg, Freiburg, Germany), as well as Gafchromic™ External Beam Therapy 3 (EBT) film (Ashland, Inc., Wilmington, DE, USA), were used and compared to arrive at a recommended standard for this critical component of small-field beam measurements. Beam profiles, including the dose fall-off region near the edge of the beam, were measured with the PTW diodes and EBT3 film and subsequently contrasted. The impact of detector physical and dosimetric characteristics on the results of the measurements was investigated and compared with film measurements. The beam penumbra was used to quantify the dose fall-off. The measurement acquired with the diodes and film showed the most significant differences in the fall-off region near the field edge. The film-based measurements clearly showed the steepest dose gradient verified by the penumbra value of 1.21 mm, followed by the SRS diode with 1.60 mm, the microSilicon diode with 1.67 mm, and the microDiamond diode with 1.83 mm. A clear correlation of each detector’s sensitive area with the penumbra was found, with the microDiamond detector at 2.2 mm diameter sensitive area having the largest penumbra, followed by the microSilicon and SRS diodes. Beam measurements for the purposes of system characterization or treatment planning system beam data acquisition depend, to a large extent, on detector characteristics. This is especially true for small-field dosimetry performed during stereotactic radiosurgery beam measurements. Careful consideration should be practiced which allows for the measurements to represent true beam characteristics and minimize the impact of the detector on the measurements. We conclude that film should be considered the reference method for such measurements with the ZAP-X due to its smallest physical measurement resolution of 23.1 µm. Potential drawbacks to this methodology are the need to calibrate the film relative to the dose and possible problems with saturation and non-linear film response for very high and very low optical densities.

## Introduction

The ZAP-X® is a new, dedicated, self-contained, and self-shielded radiosurgery system developed and manufactured by ZAP Surgical Systems, Inc., San Carlos, California. This device is intended for stereotactic radiosurgery treatment of benign and malignant intracranial and cervical spine lesions. A 3.0 megavolt (MV) S-band linear accelerator (linac) is the source of therapeutic radiation [1-2]. This radiotherapy system shares with other systems the required accuracy of dose deposition and for rapid peripheral dose fall-off. However, it utilizes very low energy (mimicking cobalt-60 (Co-60)), a very short source-to-axis distance (SAD) of 45 cm, and complete self-shielding. The latter characteristic translates into minimal facility shielding requirements but also results in a very heavy system design with a spherical treatment chamber that provides most of the needed shielding.

Due to the rapid dose fall-off, it is critical to select the correct dosimetry detector when beam data are acquired as a baseline for treatment planning purposes. Several characteristics of the detectors, such as detector area and volume, dose rate, and energy dependence, as well as the speed of detection, will determine the precision with which the beam data, and especially the beam cross profiles, can be measured. The measured data will be used in the treatment planning system of the ZAP-X for the calculation of dose and the representation of dose distribution of individual treatment plans. Therefore, it is critical that the measurement of beam data is reflecting the actual dose as delivered by the ZAP-X and the influence of the detector on the data is minimized.

This work focuses on the characteristics of various types of detectors and their influence on the measured beam data. The differences between detectors are analyzed and conclusions on the proper selection of detectors for this specialized task will be drawn. These conclusions are meant to provide guidance to the clinical medical physicist during the beam data acquisition and clinical commissioning process of the ZAP-X.

The clinical results of ZAP-X treatments are described previously [3].

## Technical report

Materials and methods

**Figure 1:**
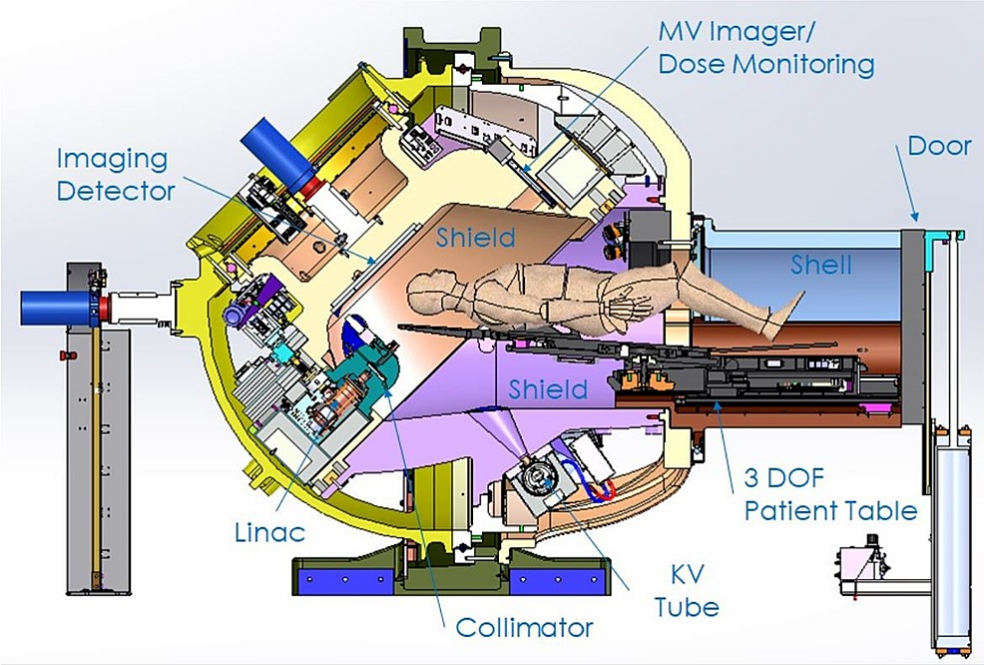
Cross-sectional view of the ZAP-X system DOF: degree of freedom; KV: kilovoltage; MV: megavoltage

The ZAP-X is shown in Figure 1 and has been described in more detail in previous publications [1-2].

The ZAP-X system has a SAD of 45 cm (Figure 1). Given the short SAD, an isocentric treatment geometry, and the fact that all imaging and shielding components rotate with the beam, the resulting treatment sphere is relatively compact.

**Figure 2:**
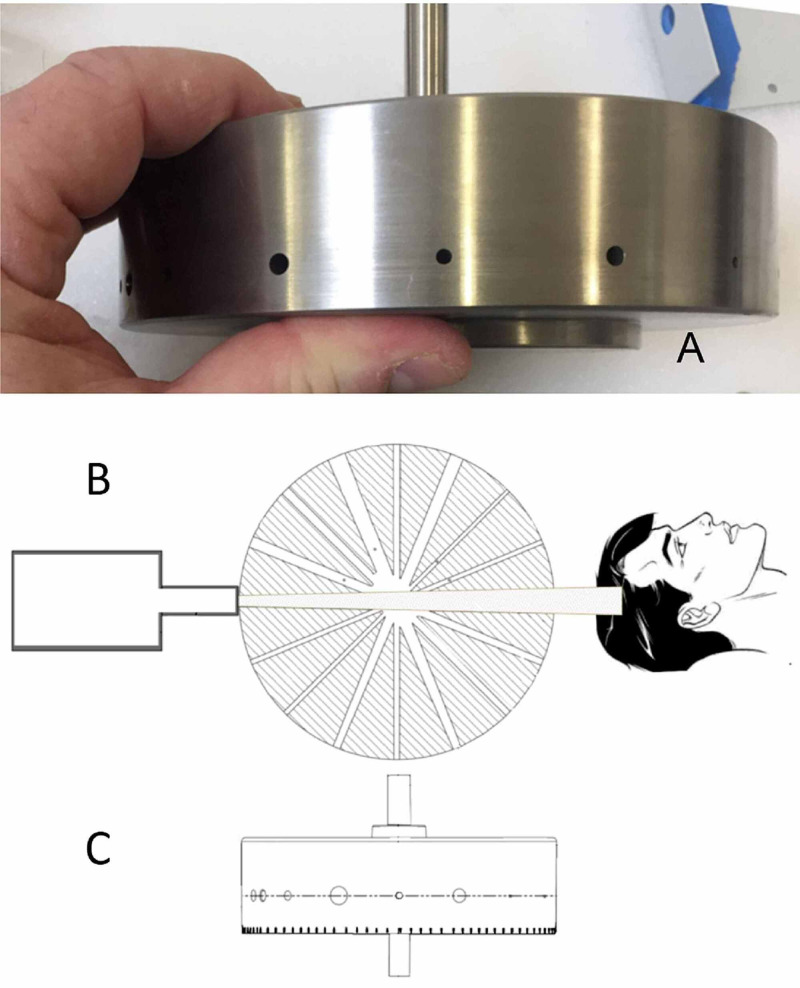
Image of the ZAP-X collimator wheel and the beam’s eye view drawing A) collimator wheel; B) a cross-sectional view of the collimator wheel; C) view along the beam central axis

A customized collimator design is critical to the overall performance of the ZAP-X system. The ZAP-X collimator consists of a shielded 15 cm diameter tungsten wheel oriented with its rotational axis perpendicular to the beam’s central axis; the goal of the design is to minimize radiation leakage and achieve a fast dose fall-off near the beam edge. Beam selection is accomplished by rotating the wheel within its tungsten-shielded housing [4]. Divergent small circular paths (apertures) are cut through the wheel and have a range of diameters (4.0, 5.0, 7.5, 10.0, 12.5, 15.0, 20.0, and 25.0 mm), as measured at the machine isocenter. Each path of the wheel traverses its diameter at different angular positions to produce the optimal collimator diameter. Figure 2A and 2C show the collimator wheel image and the beam’s eye view drawing [4]. Figure 2B shows the schematic application of the collimator wheel during the treatment process.

**Figure 3:**
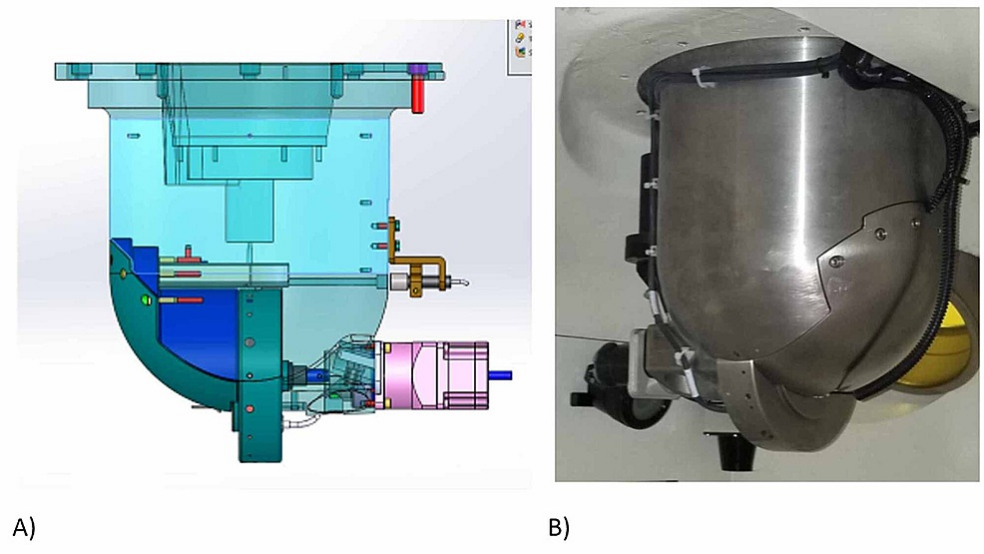
Shielded collimator housing with mounted collimator wheel A) Elevation drawing viewed in a plane with the collimator rotational axis; B) tungsten collimator housing with protruding collimator wheel.

The rotating collimator wheel is recessed into a rounded, cylindrically-shaped, solid tungsten collimator housing as shown in Figure 3.

For stereotactic radiosurgery applications, targeting accuracy and a steep peripheral dose fall-off are among the most critical characteristics. A steep dose fall-off at the edge of the beam will facilitate the precise treatment of small target volumes and simultaneously minimize the dose to nearby critical structures.

**Figure 4:**
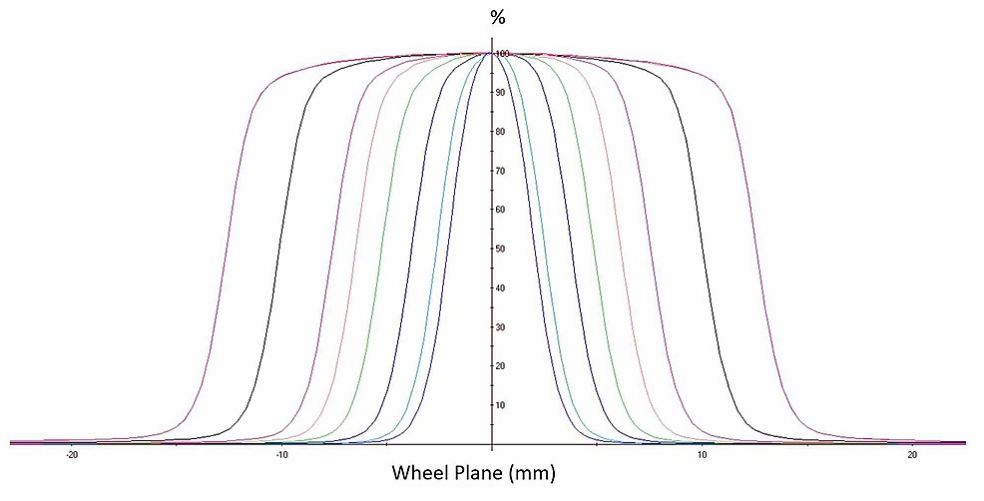
Beam dose profiles measured at the depth of maximum dose in water for all collimator sizes

Beam dose profiles were measured at a depth of maximum dose (d_max_), scanned along a direction perpendicular to the beam central axis (cax) for all collimator sizes, and are shown in Figure 4. The beam dose profiles were globally normalized to 100% on the cax for the largest 25 mm collimator field size.

**Figure 5:**
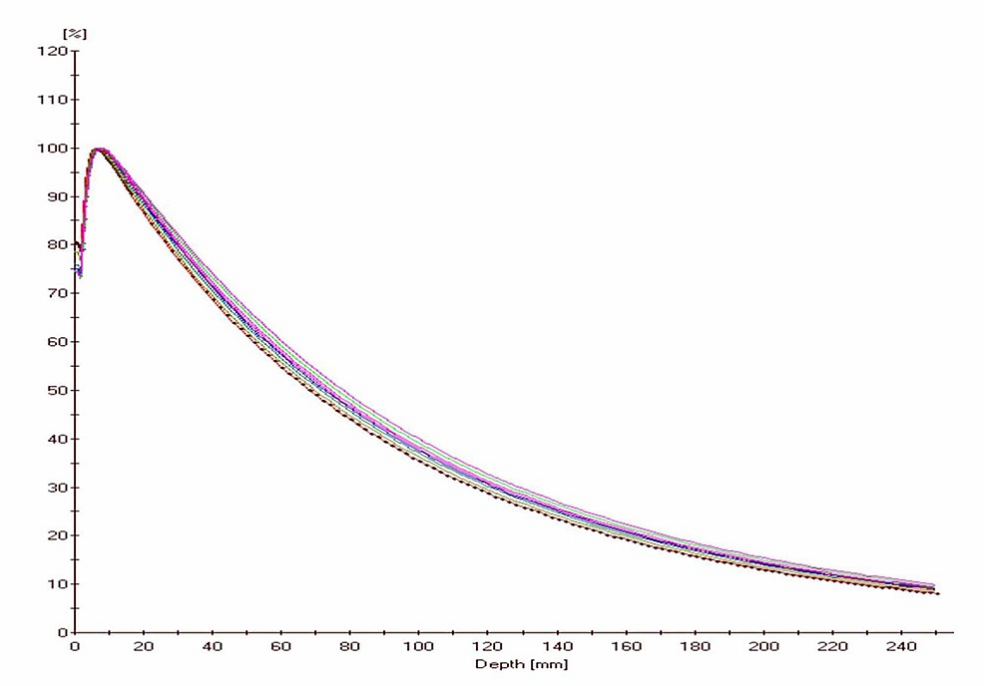
Percent depth dose measurements for all field sizes measured in water

Percent depth dose (PDD) measurements were performed for all field sizes and are displayed in Figure 5. For beam dose profiles and PDD measurements, the source-to-surface distance (SSD) was set constant to SSD = 45 cm. When accounting for the difference in SSD, the beam penetrative quality was verified to correspond to a 3 MV nominal accelerating potential [5]. 

As many of the beam quality and beam performance measurements depend on the physical and dosimetric response of the detector used, the goal of this work is to compare the differences of various detectors commonly used for small-field dosimetry and stereotactic radiosurgery treatment field analysis. Additionally, we develop a standard for measurement and will derive a universally acceptable characterization of the beam penumbra. The detectors considered include 1) Physikalische Technische Werkstaetten (PTW) micro Silicon diode (model #60023), 2) PTW micro Diamond diode (model #60019), 3) PTW Stereotactic Radiosurgery (SRS) diode (model #60018) (PTW-Freiburg, Freiburg, Germany), and 4) Gafchromic™ External Beam Therapy (EBT) 3 film (Ashland, Inc., Wilmington, DE, USA), read with a high-resolution (23.1 µm pixel) film scanner.

From a clinical perspective for intracranial and head and neck stereotactic radiosurgery, as well as stereotactic body radiotherapy, the two most critical beam performance aspects of the system are beam positioning accuracy in relation to the target volume and rapid dose fall-off near the beam edge. Rapid dose fall-off allows for aggressive treatment planning of a target volume in the direct vicinity of a critical anatomic structure, such as the trigeminal nerve treatment volume near the brainstem [6]. For a single beam profile, this dose fall-off is often quantitatively described by the beam penumbra, which is defined as the spatial distance between the 80% dose point of the beam profile and the 20% dose point when the cax value of the beam profile is normalized to 100%. For the purposes of this study, all beam dose profiles are evaluated at d_max_ = 7.0 mm.

The penumbra is determined by a geometric and a dosimetric component. The geometric component depends on the focal spot size, the source-to-detector-distance (SDD), the source-to-collimator distance (SCD), and the shape of the collimator aperture. The dosimetric component is dependent on the measurement of medium electron density and the energy composition of the therapeutic beam. The geometric penumbra will increase with the size of the focal spot size and the SDD but decrease for larger SCD. The short SAD of 45 cm, divergent collimator aperture focused on a modeled virtual beam focal position, and a small focal spot size of 1.8 mm were chosen to optimize the dose fall-off and minimize the penumbra. The dosimetric portion of the penumbra is a constant for the 3 MV photon nominal accelerating potential (NAP) in water as the measurement medium [5]. The NAP of 3 MV is optimized for treatment depths in the cranium and minimizes the out-of-field radiation scatter compared to the more commonly used 6 MV NAP with other linac-based radiosurgery devices, thereby further minimizing the beam penumbra.

In order to measure the true beam dose profile, and especially the dose fall-off, it will be critical to consider the detector characteristics listed below. Several aspects should be taken into account when selecting the most appropriate detector and scanning method. Such characteristics include the 1) volume effect and total detector area perpendicular to the cax, 2) energy dependence, 3) dose sensitivity and total detector volume, 4) speed of response and measurement position dwell time, 5) step size of the detector movement across the field, and 6) dose reproducibility and linearity.

For optimized penumbra measurements, all such characteristics will need to be considered for the selection of the most appropriate detector and measurement method for beam profile and penumbra measurements.

The physical characteristics of each of the detectors are shown in Table 1.

**Table 1:** Detector Physical Parameters

Detector	microSilicon	microDiamond	SRS diode
Diameter of sensitive volume (mm)	1.5	2.2	1.0
Depth of sensitive volume (Vol/µm)	18	2	28
Sensitive volume (mm^3^)	0.032	0.008	0.032
Density of epoxy (g/cm^3^)	1.15	1.09	1.77

**Figure 6:**
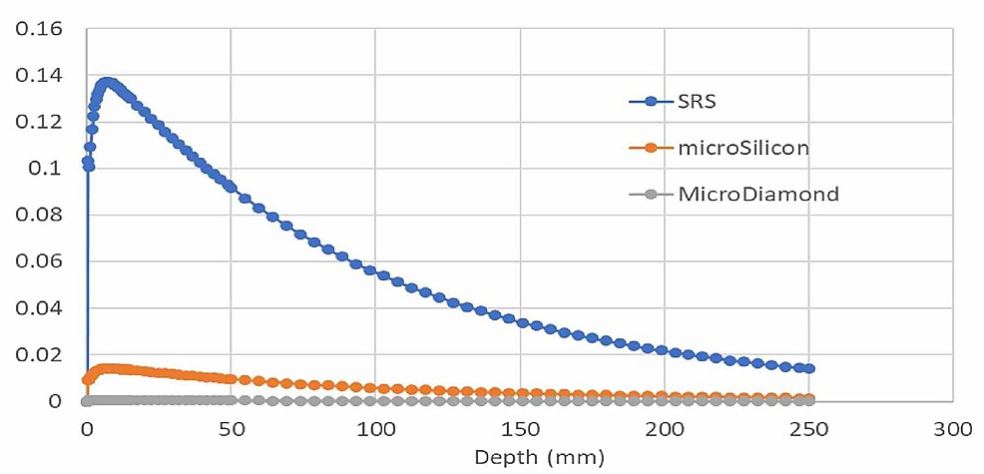
Absolute response of the SRS diode, microSilicon diode, and microDiamond diode with depth

The volume effect is expected to have a direct impact on the measurement of the penumbra, as a larger detector area is expected to increase the penumbra so that measurement results show a more gradual dose fall-off compared to the actual dose fall-off. All diode detectors used are known to be quite energy-independent. Therefore, this factor will not play a role in the measurement of the dose fall-off. The impact of dose sensitivity for the used detectors is shown in Figure 6. The amount of signal clearly depends on the total detector volume irradiated and the overall detector efficiency. The speed of the response can be considered a secondary factor. However, the speed of the beam profile scan (or point dwell time) must be optimized to capture the entire amount of radiation present at each dwell position.

**Figure 7:**
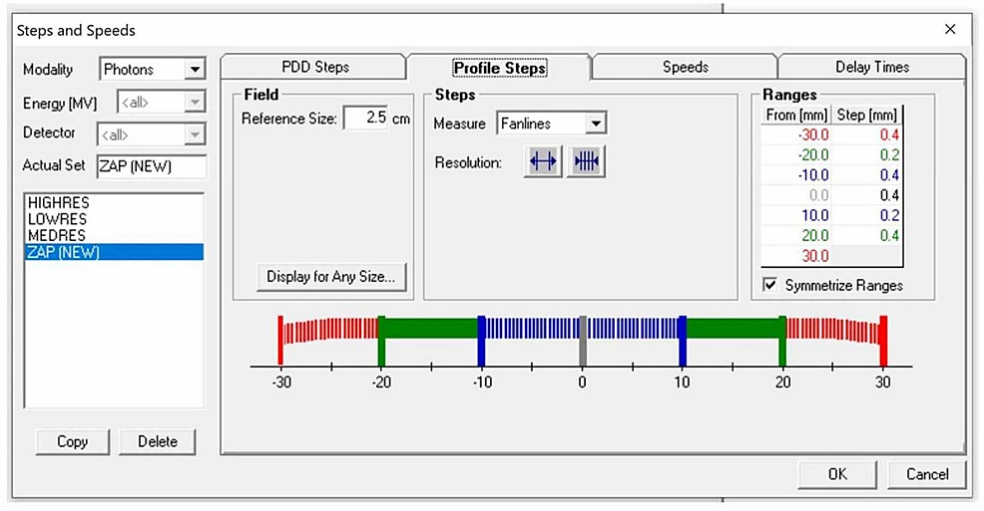
Step size definition for percent depth dose (PDD) and beam profile measurements

The step size of the beam profile was optimized and was smallest in the area of the peripheral dose fall-off and larger in the areas of low-dose gradient (Figure 7). Dose reproducibility and dose linearity (or proportionality) are known to be optimized for all three PTW semiconductor detectors, with the microDiamond diode being slightly superior to the SRS and microSilicon diodes.

The Gafchromic EBT3 film exposures were scanned with an Epson 12000 film scanner with 1100 dpi (dots per inch) and 23.1 µm spatial resolution (Epson America, Inc., Los Alamitos, CA, USA). Minor spatial scaling was applied to ensure 50% of the relative dose at the field size. The intensity profiles were then converted to dose by film calibration which relates the optical density of the film to dose. Care was applied to ensure that the dose exposures were of a magnitude that fell within the linear response range of the optical density to dose conversion curve.

The focus of this work is on the direct comparison of detector performance and not on a comprehensive beam performance evaluation which was previously performed [2]. The smallest 4 mm collimator was chosen for this direct detector comparison since this collimator represents the highest level of characterization difficulty and requires the most precision to accurately determine dose fall-off characteristics.

Results

**Figure 8:**
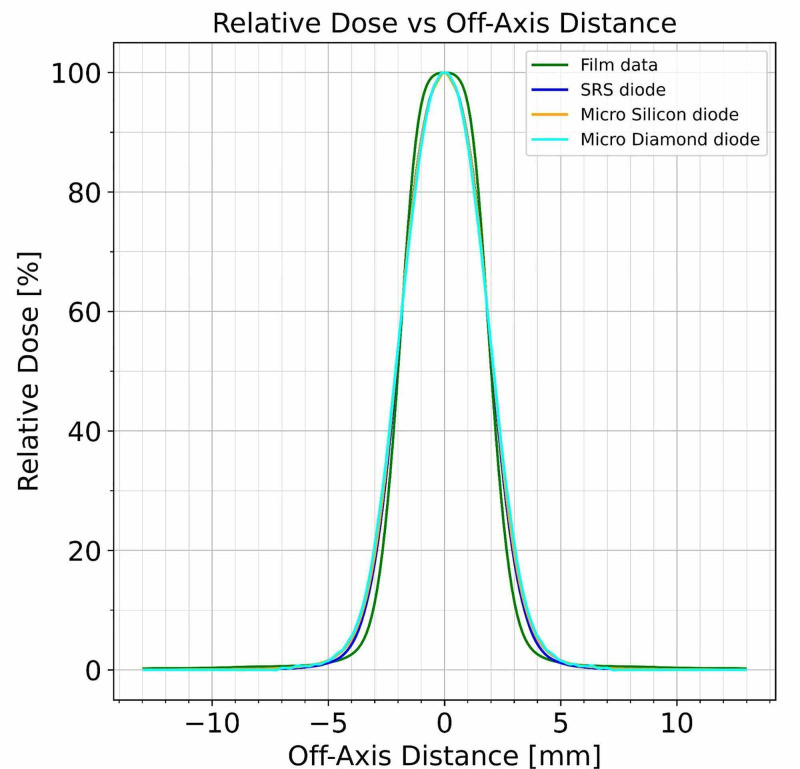
Beam profiles superimposed for three PTW detectors and film at 4 mm diameter collimator measured at the depth of maximum dose

Beam profiles were measured for the smallest collimator size of 4 mm with the described PTW diode detectors in water and with Gafchromic EBT3 film at d_max_ = 7 mm. All scans were normalized to 100% of the dose on the cax. The resulting superimposed profiles are shown in Figure 8.

**Figure 9:**
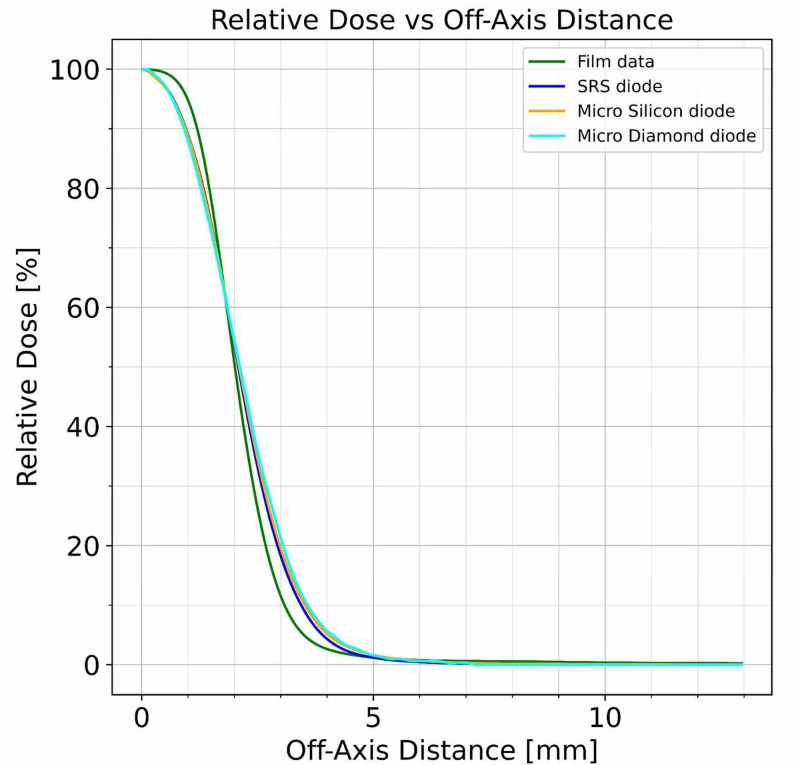
Dose measurements near the edge of the treatment field for the 4 mm collimator at the depth of maximum dose

A more detailed presentation of the dose fall-off region at the beam’s edge clearly demonstrates the effect of the detector characteristics on the measurement with the microDiamond diode showing the most gradual dose fall-off. These results are shown in Figure 9 for the 4 mm diameter collimator.

**Figure 10:**
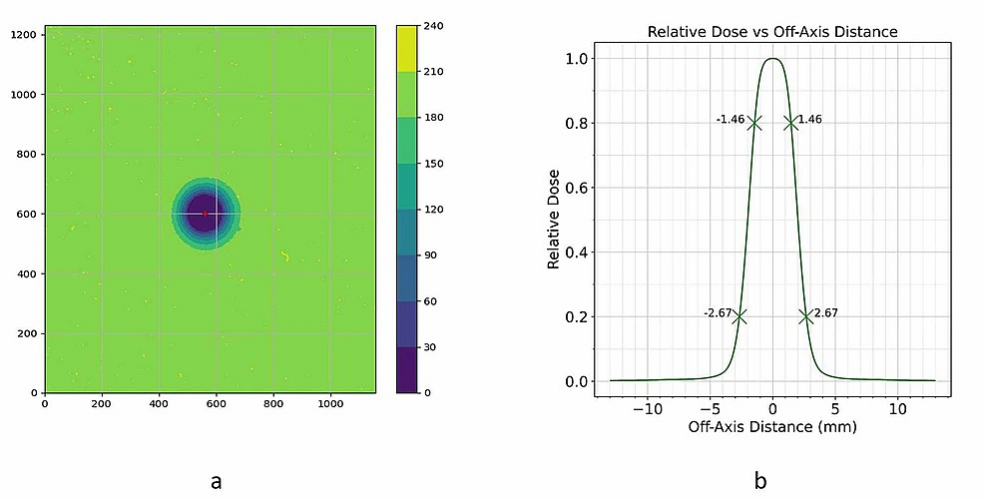
Gafchromic EBT3 film measurement results a) film exposure; b) composite beam profile

Figure 10 show the measurement results for the dose-calibrated Gafchromic EBT3 film evaluated with a film scanner: a) the film exposure and b) the composite beam profile. The composite beam profile was averaged over the entire 360-degree azimuth of the circular field size.

The evaluation of the EBT3 film measurement shows a left and right penumbra of 1.210 mm. The three described diode detectors measure significantly larger penumbras. Table 2 summarizes the penumbra findings.

**Table 2:** Comparison Chart of the Beam Penumbra for Various Detectors

Diode type	microSilicon	microDiamond	SRS diode	EBT3 film
Left penumbra (mm)	1.690	1.860	1.590	1.210
Right penumbra (mm)	1.640	1.800	1.610	1.210
Mean penumbra (mm)	1.665	1.830	1.600	1.210

The mean penumbra of the EBT3 film exposures was found to be at least 0.39 mm smaller than any of the diode detectors, with the largest deviation to the microDiamond at 0.62 mm. These differences are considered significant for a 4 mm collimated field size.

**Figure 11:**
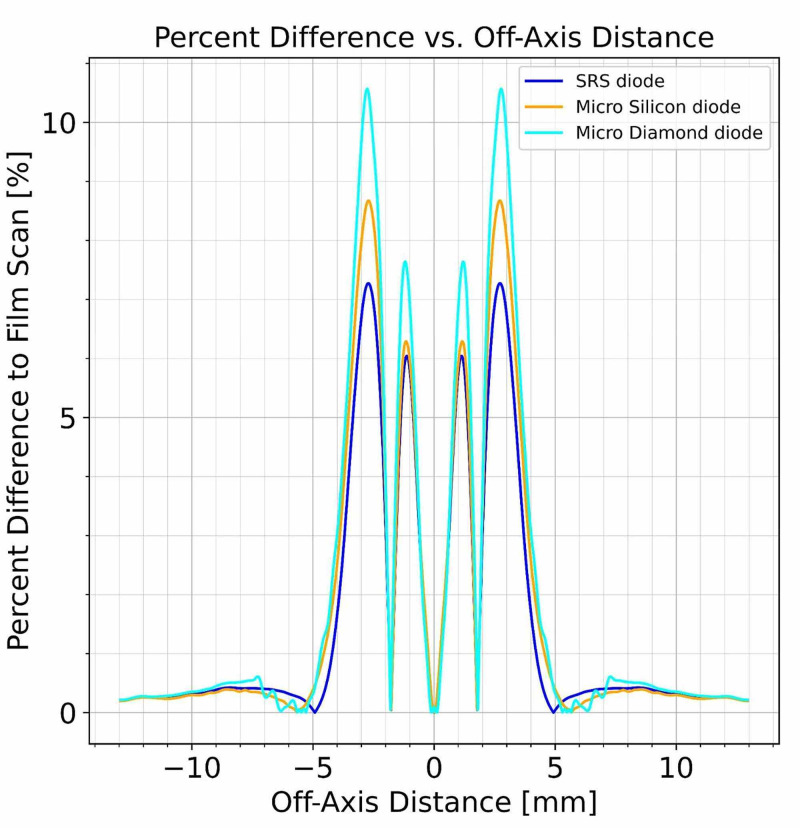
Percent dose difference for each diode compared to the EBT3 film measurements

Additionally, the normalized beam profiles of the SRS diode, the microSilicon diode, the microDiamond diode, and the EBT3 film exposures were quantitatively compared. The percentage dose difference for each of the three diode detector scans compared to the EBT3 film scan was plotted in Figure 11.

The EBT3 film exposure measurements and dose evaluation show the most rapid dose fall-off for the 4 mm collimator beam profile measured as penumbra (80% to 20% dose point positions). As the PTW microSilicon, microDiamond, and SRS diodes show significantly larger penumbra values for the same beam (as seen in the increase of the difference at the beam’s edge), it can be concluded that film-based measurements scanned with a spatial resolution of 1,100 dpi represent the most realistic penumbra measurements.

## Discussion

The ZAP-X, like the GammaKnife and CyberKnife is a dedicated radiosurgery device. Applicability and beam characteristics are more aligned with the GammaKnife, despite the difference in radiation source. The 3 MV nominal accelerating potential, a short SAD, and the very small beam penumbra make the ZAP-X system dosimetrically quite comparable with the GammaKnife.

It is the responsibility of the clinical medical physicist on record for an institution implementing the ZAP-X, or any other radiotherapy device, to evaluate the best possible measurement technique and equipment for dose profile beam performance evaluation. As part of this consideration, detector selection is critical to accurately measure dose fall-off at the periphery of the beam and to ensure that the acquired beam data accurately presents true beam characteristics. Film measurement has been shown to be the most accurate method and should be employed whenever the highest level of accuracy is required.

For a complete set of beam data measurements, as needed for delivery system characterization and treatment planning system commissioning, using film-based measurements with its need for dose calibration and scanning of each film might prove impractical. Instead, it is recommended to use the smallest available sensitive area diode detector with energy and dose rate independence. The known increase in penumbra due to the measurement technique should be taken into account during the process of treatment planning system commissioning, possibly by film results-based adjustments of the diode measured beam profiles.

It is noted that only the 4 mm collimator of the Zap-X was evaluated here, representing the smallest available field size for any commercially available linac-based radiotherapy system. The significance of detector-dependent differences will have a slowly diminishing effect for increasing field sizes, and the stated findings and conclusions shall be applied to the smallest field sizes of less than a 10 mm diameter only.

## Conclusions

Film-based dose measurements have been shown to be the most accurate method of measuring stereotactic radiosurgery very small-field beam performance, especially in the fall-off region at the edge of the beam. We recommend employing film as the reference measurement technique for all ZAP-X collimators. Additionally, we recommend against the use of the microDiamond detector for dose profile measurements of the smallest ZAP-X field sizes (4 mm, 5 mm, 7.5 mm) due to its relatively large sensitive area.
